# Supplementary material for: Evidence supported by Mendelian randomization: impact on inflammatory factors in knee osteoarthritis
Source: Front Med (Lausanne). 2024 May 28;11:1382836. doi: 10.3389/fmed.2024.1382836 (PMC11165061; doi:10.3389/fmed.2024.1382836)
Supplement: Supplementary file 1 [file Data_Sheet_1.ZIP › data/GCST90274770_5e6/770.leaveoneout.pdf]

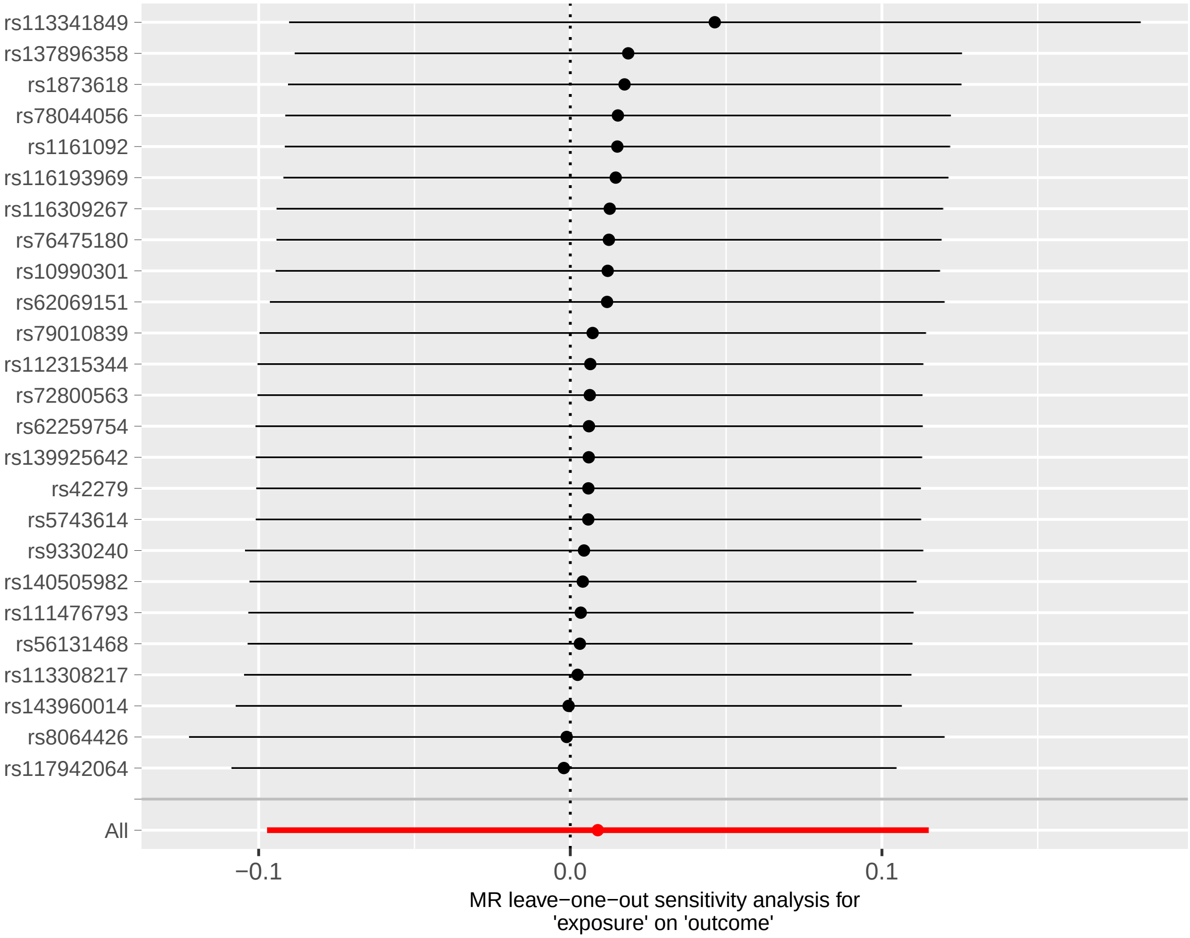
rs113341849
rs137896358
rs1873618
rs78044056
rs1161092
rs116193969
rs116309267
rs76475180
rs10990301
rs62069151
rs79010839
rs112315344
rs72800563
rs62259754
rs139925642
rs42279
rs5743614
rs9330240
rs140505982
rs111476793
rs56131468
rs113308217
rs143960014
rs8064426
rs117942064
All
−0.1
0.0
0.1
MR leave−one−out sensitivity analysis for
'exposure' on 'outcome'
